# Persistent Hypoglossal Artery

**DOI:** 10.5334/jbsr.1481

**Published:** 2018-02-20

**Authors:** Bruno Coulier

**Affiliations:** 1 Clinique Saint-Luc, Bouge, BE

**Keywords:** Persistent primitive hypoglossal artery, CT angiography, Anatomic variant

## Case Report

A 47-year-old woman was referred to the imaging department for Computed Tomography Angiography (CTA) of the supra-aortic arteries in order to investigate the cause for systemic blood pressure asymmetry. Prior Doppler ultrasonography of the cervical arteries had failed to demonstrate the vertebral arteries.

**Figure 1:**
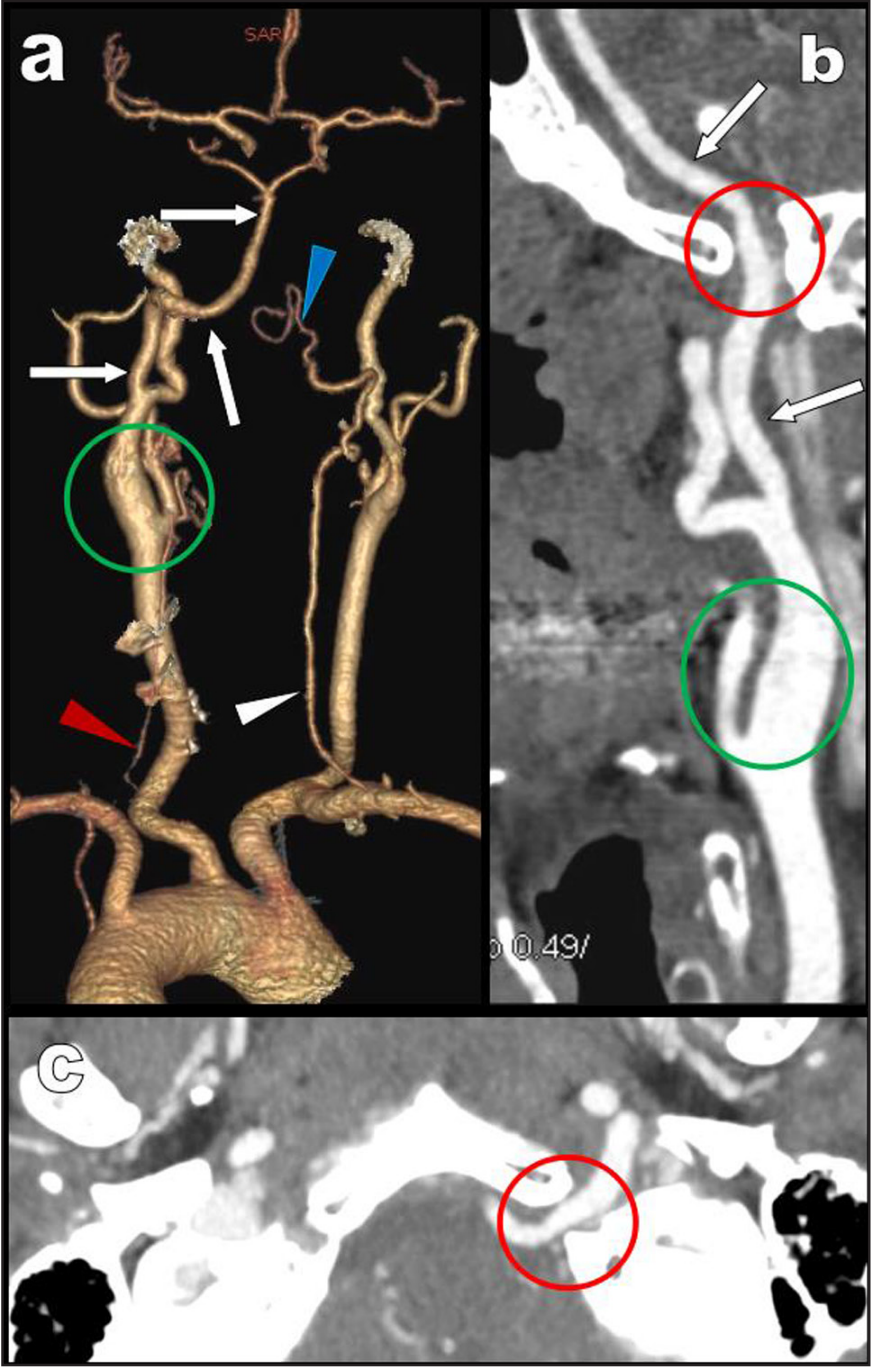
CTA: **a)** posterior-anterior VR view show major hypoplasia of the vertebral arteries. They are not connected to the basilar trunk which appear only feed by a large ascending artery emerging from a large left internal carotid. Curvilinear reconstruction **(b)** and axial MIP view **(c)** at the level of the foramen magna show this large hypoglossal artery penetrating the skull through the supracondylar hypoglossus canal.

**Figure 2:**
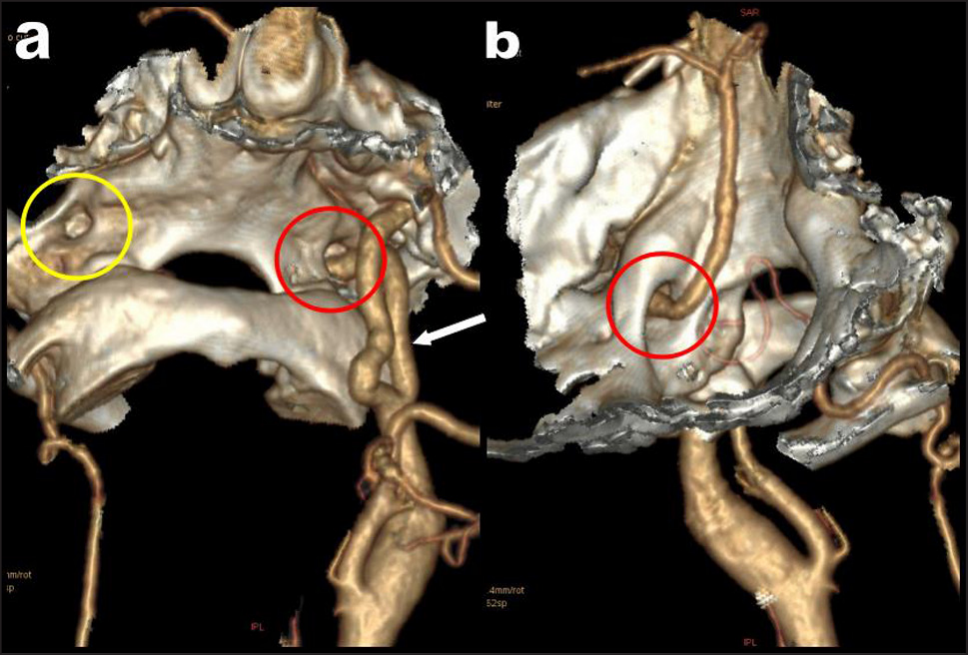
3D volume rendering views with bone structures showing the hypoglossal artery penetrating the skull through the supracondylar hypoglossal fossa.

CTA (Figure 1) confirmed major hypoplasia of the vertebral arteries (red and white arrowheads on a) especially on the left side (red arrowhead). These hypoplastic vertebral arteries were not connected to the basilar artery (blue arrowhead on a) which was only fed by a large ascending artery (white arrows on Figures 1 and 2) emerging from a large left internal carotid artery (green circle). 3D volume rendering views without bone removal (Figure 2a and b) showed that this atypical artery was penetrating the skull through the hypoglossus canal (red circle on Figures 1 and 2). The artery was clearly identified as a persistent hypoglossal artery. The finding was considered as fortuitous.

## Comment

**Figure 3:**
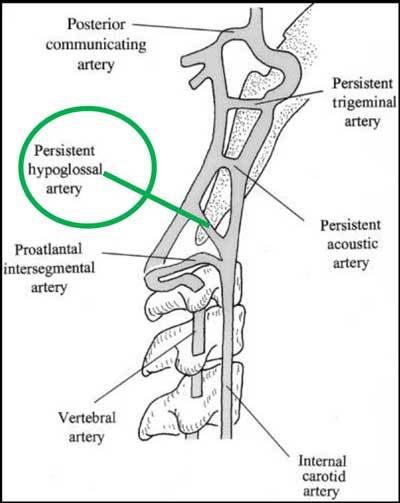
Schematic representation of the different fetal anastomoses between the vertebral arterial system and the carotid arterial system.

During embryology, the primitive forebrain is supplied by the carotid system. Two other parallel longitudinal neural arteries form and run along the surface of the primitive hindbrain and may fuse to create the basilar artery. These arteries are initially supplied by the carotids through four important anastomoses (Figure 3) comprising from top to bottom the trigeminal artery, the otic artery (passing through the internal auditory canal), the hypoglossal artery (HA) (passing through the hypoglossal canal) and the pro-atlantal segmental artery (passing through the foramen ovale) (Figure 3). After formation of the posterior communicating artery, which develop between the distal internal carotid and the ipsilateral longitudinal neural artery the other anastomoses will regress and disappear.

The last anastomosis to disappear is the pro-atlantal artery which continues to supply the hind brain until the vertebral arteries develop and take over.

Failure of obliteration of these fetal anastomoses results in the persistence of one or more of these embryonic arteries which may lead to hypoplasia of the vertebrobasilar system (especially the vertebral arteries) as shown in the reported case. The ipsilateral vertebral artery may be completely absent.

The HA is more frequent in females and on the left side. Although the HA is the second most frequent persistent anastomosis after the trigeminal artery, it only has a very low incidence of 0.1–0.2% [1]. Bilateral HA are extremely rare. The HA usually arises from the internal carotid (type 1) at the level of C1–C3 and more rarely from the external carotid artery (type 2). HA is an anatomic variant and typically presents as an incidental finding on imaging studies. Although HA is usually asymptomatic, it may be associated with intracranial aneurysms especially at the junction of the HA with the basilar artery in 26–33% of cases. Glossopharyngeal neuralgia and hypoglossal palsy are possible symptoms. HA may also constitute a risk for ischemic or embolic infarction to the posterior cerebral territories especially in patients presenting with proximal carotid stenosis.

The key finding to distinguish the persistent hypoglossal artery from the pro-atlantal artery that has nearly the same orientation is the identification of the foramen through which the artery passes. This may be better achieved by CT and/or MRI than by conventional angiography.

This artery is important to clearly identify before carotid endarterectomy or skull base surgery. Injury, thrombosis or embolism can lead to catastrophic ischemia in the posterior cerebral territory of which the HA constitutes the sole arterial supply.
